# Liquid biopsy in biliary tract cancers: early diagnosis, precision therapy, and prognostic evaluation

**DOI:** 10.3389/fonc.2025.1705162

**Published:** 2025-12-19

**Authors:** Guanhua Wu, Xinjian Xu, Honghua Zhang, Li Peng, Chao Liu

**Affiliations:** 1 Department of Biliary-Pancreatic Surgery, Sun Yat-sen Memorial Hospital, Sun Yat-sen University, Guangzhou, China; 2 Guangzhou Key Laboratory of Precise Diagnosis and Treatment of Biliary Tract Cancer, Sun Yat-sen Memorial Hospital, Sun Yat-sen University, Guangzhou, China; 3 Guangdong Provincial Key Laboratory of Malignant Tumor Epigenetics and Gene Regulation, Guangdong-Hong Kong Joint Laboratory for RNA Medicine, Sun Yat-sen Memorial Hospital, Sun Yat-sen University, Guangzhou, China; 4 Department of Hepatobiliary and Pancreatic Surgery, The Fifth Affiliated Hospital of Xinjiang Medical University, Urumqi, China; 5 Medical Research Center, Sun Yat-Sen Memorial Hospital, Sun Yat-Sen University, Guangzhou, China

**Keywords:** biliary tract cancers, early diagnosis, liquid biopsy, precision therapy, prognostic evaluation

## Abstract

Biliary tract cancers represent a heterogeneous group of malignancies, including gallbladder cancer, intrahepatic cholangiocarcinoma, perihilar cholangiocarcinoma, and distal cholangiocarcinoma. These cancers share several common characteristics, such as challenges in early diagnosis, aggressive biological behavior, limited treatment options, and a propensity for drug resistance, all of which contribute to a notably poor prognosis for patients. Therefore, discovering new tumor biomarkers is urgently needed for early diagnosis, personalized treatment, and the prediction and monitoring of treatment effectiveness. Given that biopsy procedures are invasive and that repeated biopsies can diminish patient compliance, the ongoing advancement of liquid biopsy technology—characterized by its non-invasiveness and accessibility—promises to usher in a new epoch in clinical oncology. Currently, liquid biopsy mainly focuses on detecting and analyzing circulating tumor cells, cell-free DNA, and extracellular vesicles. This review aims to summarize the latest research advancements and future clinical applications of liquid biopsy in the context of biliary tract cancers.

## Introduction

Biliary tract cancers (BTCs) represent the second most prevalent category of primary hepatobiliary malignancies, constituting approximately 3% of all digestive system tumors (1). BTCs can be classified based on anatomical location into four categories: gallbladder cancer (GBC), intrahepatic cholangiocarcinoma (iCCA), perihilar cholangiocarcinoma (pCCA), and distal cholangiocarcinoma (dCCA). Currently, the only potentially curative option is surgical resection, but the early symptoms are often unnoticed, leading to advanced stage diagnoses and missed surgical opportunities. Although the etiologies of GBC and cholangiocarcinoma (CCA) differ, both are closely linked to the long-term interplay of genetic susceptibility, chronic inflammation, congenital anatomical abnormalities, and environmental factors, among which chronic inflammation is regarded as the primary driving force (2). Because there are no effective early diagnostic techniques, only about 10% of GBC patients are eligible for radical resection, with most being diagnosed with locally advanced disease or distant metastasis (3), leading to a median overall survival is only 6 months with a 5-year survival rate under 5%. In contrast, radical resection can improve the 5-year survival rate to between 21% and 69% (4, 5). Similarly, CCAs are typically asymptomatic in early stages. Consequently, most patients present with metastatic or locally advanced disease, rendering them ineligible for resection; only about 25% are candidates for curative surgery at diagnosis (6). Post-resection, the 5-year survival rates remain dismal, ranging from 22–44% for iCCA, 11–41% for pCCA, and 27–37% for dCCA (7). These sobering statistics collectively underscore the critical need for early diagnosis to improve patient prognosis.

However, current imaging techniques exhibit significant limitations in the detection of early-stage BTCs. Early GBC often cannot be reliably identified, with some cases being discovered incidentally during postoperative pathology, a phenomenon referred to as ‘incidental GBC’. CCA is frequently diagnosed when jaundice presents; however, by this stage, most patients have already progressed to locally advanced disease. Furthermore, early detection efforts for high-risk populations like individuals with cholelithiasis, sclerosing cholangitis, or pancreaticobiliary maljunction are primarily in the exploratory phase globally. Despite the recommendation for biopsy to obtain tissue for diagnosis in patients ineligible for curative-intent surgery, establishing a definitive diagnosis in BTCs remains challenging. Key obstacles include poor tumor accessibility—particularly in the perihilar region—and a considerable biopsy failure rate reported to be as high to 27% (8). The limitations of conventional sampling are further underscored by the low sensitivity (20–40%) of biliary cytology (9). These diagnostic challenges have accelerated the development of non-invasive alternatives over the past decade. Among them, liquid biopsy has gained considerable interest as a complementary tool, a trend further catalyzed by the procedural restrictions during the COVID-19 pandemic (10). As a non-invasive and efficient diagnostic technique, it has emerged with remarkable potential for clinical application. It facilitates early screening before imaging can detect lesions, particularly in high-risk populations, and enables the timely identification of potential malignancies. This advancement allows patients to undergo surgical interventions at an earlier stage, significantly improving survival rates, reducing disease burden, and conserving medical resources.

For patients with locally advanced BTCs, neoadjuvant chemotherapy is regarded as a potential conversion treatment strategy. However, responses to chemotherapy regimens exhibit significant variability among individuals. Even in patients who successfully undergo surgery, metastasis may still occur either intraoperatively or within a short period following the operation. For those who miss the surgical opportunity, treatment primarily depends on chemotherapy, targeted therapy, and immunotherapy; nevertheless, drug resistance continues to pose a significant clinical challenge. Liquid biopsy offers significant advantages in precision therapy and the dynamic monitoring of treatment responses. It may help avoid ineffective therapies, reduce drug-related side effects, lower medical costs, and provide evidence for timely adjustments in treatment strategies. Furthermore, prognostic assessment is crucial for treatment planning in BTCs. By utilizing liquid biopsy technology, potential candidates for neoadjuvant therapy can be identified in advance. In cases of advanced-stage disease, early prediction of chemotherapy resistance and prognosis allows for more tailored and sensible clinical decision-making.

For those diagnosed with BTCs and unable to undergo radical resection, tissue biopsy is the diagnostic ‘gold standard,’ but it is a highly invasive procedure that risks tumor seeding along the needle tract. In addition, false-negative results may occur due to inaccuracies in targeting or inadequate sampling. Conversely, liquid biopsy has attracted significant interest because it is non-invasive, highly sensitive, and allows for repeated sampling. Liquid biopsy has recently demonstrated considerable promise for early diagnosis, personalized treatment, and monitoring therapy responses in patients with tumors and other diseases by identifying biomarkers in bodily fluids like blood, bile, and urine. The biomarkers are chiefly composed of three categories: circulating tumor cells (CTCs), cell-free DNA (cfDNA), and extracellular vesicles (EVs) (11–14). In the realm of BTCs, current research predominantly emphasizes biomarker detection in both blood and bile. The aim of this article is to review the current advancements in research and the clinical application of liquid biopsy for early diagnosis, precision treatment, and prognosis evaluation of BTCs.

## The methods and development of liquid biopsy

Currently, EVs, CTCs, and cfDNA have emerged as the three primary components of liquid biopsy (15). In 1967, Wolf captured the first electron micrographs of EVs (16). Subsequently, in 1996, Raposo demonstrated the biological activity of EVs, revealing that EVs derived from immune cells are capable of antigen presentation (17). EVs are membrane-bound structures emitted by different cell types, including tumor cells. Based on their formation process and dimensions, these diverse membrane structures are categorized as exosomes and microvesicles. EVs contain a wide range of biomolecules, which encompass nucleic acids like DNA, mRNA, miRNA, and non-coding RNA, in addition to proteins, lipids, and numerous metabolites (18). EVs have the capacity to engage with neighboring cells, and their molecular makeup can either worsen or improve the characteristics of cancer (19). For instance, CCA cells release exosomal miR-182/183-5p into bile, which suppresses hydroxyprostaglandin dehydrogenase, enhances the secretion of PGE2 and VEGF-A, and triggers *PTGER1* activation to preserve tumor stemness, thus creating a mechanism for self-sustained progression that is dependent on bile exosomes (20). The cargo carried by EVs accurately reflects the characteristics of their parent cells (21). Research has shown that analyzing individual EV allows for the highly sensitive identification of *KRAS* mutations in pancreatic cancer, as well as the levels of *HER2* expression in breast cancer. This ability not only supports early detection but also encourages the advancement of tailored treatment approaches informed by the molecular profiles of EVs (22, 23). With ongoing technological breakthroughs and continuous methodological refinement, the emergence of high-throughput characterization platforms is anticipated. Recent developments in high-resolution imaging, microfluidics, and nanopore sensing have significantly improved the ability to detect and study individual EVs (24). Consequently, the analysis of individual EV is set to revolutionize approaches to cancer diagnosis and monitoring, providing new opportunities for early detection and enhanced patient outcomes (25).

However, the further clinical application of exosomes has been greatly hindered by the lack of reliable methods for high-quality isolation and component characterization (15). In liquid biopsy, the main limitations of EVs include their complex origins, pronounced heterogeneity, challenges in isolation and standardization, dilution of tumor-specific signals, and insufficient clinical validation, all of which collectively restrict detection sensitivity and specificity (26).

CTCs are regarded as indicators of remote metastasis in certain cancer patients (27). The first account of CTCs traces back to 1869, when Thomas Ashworth, an Australian doctor, discovered cancerous cells that looked similar to those of the original tumor within the blood vessels of a patient who had undergone an autopsy due to metastatic cancer (28). Following this, CTCs have been extracted from individuals with different forms of cancer. They are acknowledged not only for deepening our comprehension of tumor development and spread but also for their significance in evaluating prognosis, monitoring recurrence, assessing treatment efficacy, and investigating mechanisms of drug resistance (29–31). CTCs are exceedingly rare, with an estimated frequency of only 1 per 10^6^–10^7^; leukocytes, making their isolation technically challenging. Reduzzi, C. et al. found that in BTCs, a single-cell assay for detecting CTCs enabled the identification of both epithelial CTCs and non-conventional CTCs that lack epithelial and leukocyte markers. This advancement consequently resulted in an increased positivity rate for CTCs (32). Notably, reagent-free detection of PD-L1 expression on CTC surfaces shows potential for evaluating immunotherapy response and predicting clinical progression (33).

However, detection rates correlate with disease stage: sensitivity remains relatively low in early-stage cancer, whereas higher CTC counts are typically observed in advanced disease (34). Despite their promising role in cancer diagnosis and treatment, the clinical utility of CTCs is limited by several factors. Inadequate sensitivity of current detection technologies, compounded by the downregulation of surface markers in some tumors, contributes to false-negative results (35). Furthermore, CTCs exhibit significant heterogeneity, comprising subpopulations with diverse phenotypic and genetic characteristics. This variability complicates the capture of a representative subset that accurately reflects the entire tumor, thereby hindering the development of targeted therapies (36). Additionally, the frequent dormancy of CTCs—a state in which they remain metabolically inactive and do not proliferate—poses further challenges for their application in monitoring treatment responses (37). To overcome these limitations, it is essential to develop technologies that are both more sensitive and selective for isolating and characterizing CTCs, as well as to enhance our understanding of CTC biology.

cfDNA is defined as DNA that is not enclosed within a cell, and it can be extracted from plasma. In individuals without health issues, cfDNA from normal cells in the plasma is typically present in low concentrations, averaging between 10 and 15 ng/ml (38). Circulating tumor DNA (ctDNA), which is a form of cfDNA, is made up of small nucleic acid fragments that are released from apoptotic or necrotic tumor cells, or through EVs. This DNA contains genetic information that is particular to cancer cells (39, 40). Research carried out by Leon and colleagues in 1977 demonstrated that the levels of plasma cfDNA were markedly higher in cancer patients than in those who are healthy, suggesting a link between cfDNA and the existence of tumors (41). In addition, researchers first identified KRAS mutations in the blood cfDNA of pancreatic cancer patients using PCR technology in 1994, and these results aligned with those found in tumor tissue (42). The cfDNA is an ideal analyte for detecting tumor-specific mutations, copy number variations (CNVs), and integrated viral sequences. In the context of liquid biopsy, the epigenetic profiling of cfDNA has emerged as a major focus in oncology research. This encompasses various aspects, including methylomics (5mC and 5hmC), fragmentomics (fragment size, end motifs, and nucleosome positioning), and nucleosomics (histone modifications and occupancy) (43, 44). Studies indicate that the epigenetic features of cfDNA exhibit tissue-specific patterns, thereby facilitating the identification of its tissue of origin. By comparing these profiles with reference methylation databases derived from both normal and malignant tissues, the source of cfDNA can be accurately inferred (45). Machine learning (ML) models utilizing cfDNA methylation have been developed to effectively predict cancer types of unknown primary origin, demonstrating a high concordance with clinical diagnoses (46). These approaches rely on large-scale datasets to develop models with enhanced specificity and sensitivity for clinical use (47). The use of ML and artificial intelligence (AI) to combine multi-dimensional characteristics of cfDNA has greatly progressed the field of liquid biopsy applications.

In patients with cancer, ctDNA typically constitutes only about 0.01–5% of total cfDNA (48). This already small fraction can be even lower in early-stage disease due to minimal tumor shedding, potentially dropping below the detection limit of current assays and leading to false-negative results. Furthermore, biological and technical factors compound this problem. Biologically, uneven tumor shedding may prevent liquid biopsies from fully capturing spatial heterogeneity, while clonal hematopoiesis (CHIP) can introduce non-tumor variants, leading to false positives. Technically, a lack of standardization in pre-analytical and analytical workflows across laboratories hampers data reproducibility and comparability (49).

## Liquid biopsy for biliary tract cancers: blood or bile

**Figure 1:**
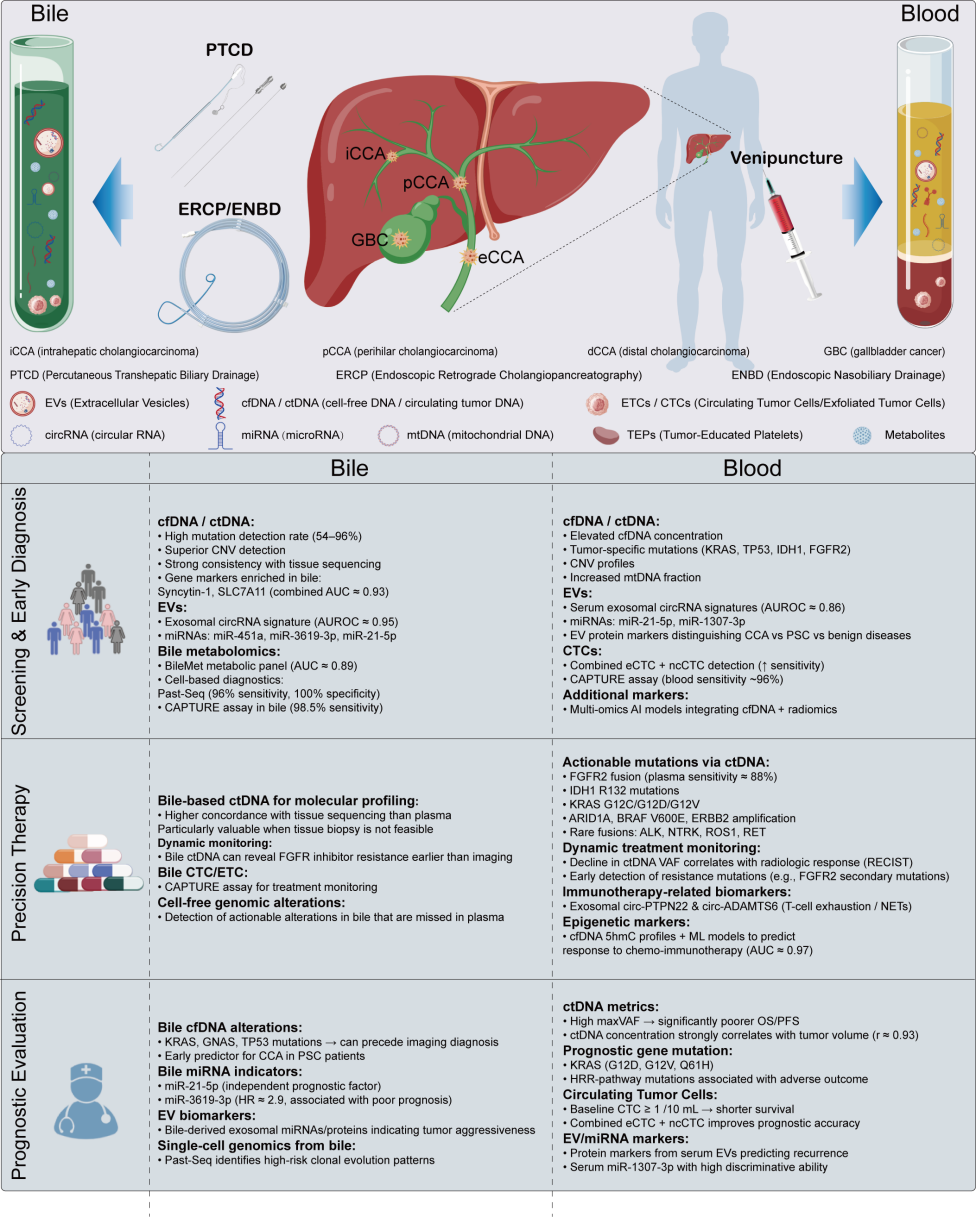
Liquid biopsy in BTCs patients. EVs (Extracellular Vesicles);cfDNA(cell-free DNA);ctDNA(circulating tumor DNA);ETCs (Exfoliated Tumor Cells);CTCs (Circulating Tumor Cells);circRNA(circular RNA);miRNA(microRNA);mtDNA (Mitochondrial DNA);TEPs (Tumor-Educated Platelets). EVs, ctDNA, ETCs, circRNA, miRNA, mtDNA and Metabolites can all be detected through liquid biopsy using bile samples. EVs, cfDNA/ctDNA, CTCs, circRNA, miRNA, mtDNA, TEPs and Metabolites can be identified in blood samples collected via liquid biopsy.

More than 95% of BTCs originate from the biliary epithelium (50). Due to their close association with the bile duct, tumors within the biliary system modify small molecules in the bile, which includes cells, EVs, nucleic acids, proteins, and metabolites that may act as potential biomarkers (51, 52). In addition, metabolites, proteins, nucleic acids, and vesicles released into the bloodstream via necrosis, apoptosis, or active secretion by tumor cells also carry cancer-related information (53). Liquid biopsy for BTCs typically requires the collection of approximately 10 mL of blood or bile and has gained considerable attention as a minimally invasive approach for assessing systemic tumors in patients, including applications in early screening and diagnosis, precision therapy, and prognostic evaluation (**Figure 1**). Furthermore, studies have revealed that urinary exosomal RNAs in patients with CCA demonstrate considerable diagnostic potential (54). Currently, plasma-based liquid biopsy is relatively well-established and is not restricted by cancer type. However, bile, as a biofluid in direct contact with tumors, is considered to have greater potential specifically for BTCs. In terms of sample collection, plasma is more easily accessible, whereas bile must be obtained through invasive procedures such as percutaneous transhepatic cholangiography (PTCD) or endoscopic nasal biliary drainage (ENBD), which limits its widespread use, particularly in early cancer screening. In summary, both blood and bile have distinct advantages and limitations in liquid biopsy for BTCs. (**Table 1**).

**Table 1:** Comparative the feature of bile and blood for liquid biopsy in BTCs.

Feature	Bile	Blood
Sample collection	Technically difficult; requires invasive procedures such as ERCP or PTCD	Easy and minimally invasive (venipuncture)
Invasive nature	Highly invasive; not suitable for routine screening	Minimally invasive; suitable for routine screening and serial monitoring
Early screening potential	Limited by invasiveness; feasible mainly in high-risk or symptomatic patients	Good for population-level or repeated screening
ctDNA concentration	High, due to proximity to tumor and direct shedding into bile	Low, because of dilution in systemic circulation
Mutation abundance	High; bile cfDNA shows enriched tumor-specific mutations	Low; tumor signals may be masked by normal cfDNA

ERCP, Endoscopic Retrograde Cholangiopancreatography; PTCD, Percutaneous Transhepatic Cholangiography and Drainage; CHIP, Clonal Hematopoiesis of Indeterminate Potential.

Technically, methods for isolating cells, extracting ctDNA, and enriching EVs from bile have matured (55). Bile ctDNA remains stable under specific conditions—up to 10 days at room temperature in cfDNA storage tubes and up to 2 months at 4 °C (56) Similarly, proteins from bile maintain stability for molecular analysis for up to 7 hours at room temperature (57). Regarding tumor signal intensity, studies have shown that bile cfDNA levels are generally higher than those in plasma due to its proximity to the tumor site, resulting in higher sensitivity and specificity (58). For instance, one study demonstrated that the concentration of cell-free nucleic acids in bile exceeded that in plasma. Using a multi-gene panel on 17 CCA patients, the detection rate of driver gene mutations was significantly higher in bile cfDNA (54%) than in plasma (17%) (59). Another study involving 30 GBC patients compared mutations in bile and tumor tissue using a 49-gene panel alongside cytology. The concordance rate of mutations between bile and tissue samples was found to be 85.7%, with the diagnostic sensitivity of ctDNA from bile (58.3%) exceeding that of cytology (45.8%) (60). Genomic analysis of bile liquid biopsy also showed higher sensitivity (46.5%) compared to cytology alone (27.9%) (61). Targeted deep sequencing of ctDNA from bile and tumor DNA showed a high sensitivity of 94.7% and a specificity of 99.9%. Moreover, the sensitivity and specificity for identifying CNVs were found to be 75.0% and 98.9%, respectively (62). In addition, the diagnostic capabilities of miR-21-5p were found to be more effective in bile (AUC = 0.913) than in serum (AUC = 0.628) and carbohydrate antigen 19-9 (CA19-9) (AUC = 0.793) (63). The initial findings emphasize the potential of bile cfDNA as a liquid biopsy method for patients with BTCs (**Table 2**). Despite these advantages, bile-derived ctDNA faces several practical challenges. Bile collection necessitates invasive procedures such as ERCP, ENBD, or PTCD, limiting its suitability for widespread or repeated testing. Moreover, the absence of standardized protocols for bile collection, processing, and storage contributes to study-to-study variability and impedes translation into real-world clinical practice. Comparative studies between bile cfDNA and tissue-based genomic profiling remain limited; although small-scale analyses have revealed high concordance, larger prospective studies are still needed for validation.

**Table 2:** Diagnostic capability of bile and blood for liquid biopsy in BTCs.

References	Biomarkers/research objectives	Bile	Blood
Wen et al (71)	circRNA for diagnostic performance	AUC = 0.947	AUC =0.861
Tsai, Y. C. et al (81)	ETCs/CTCs for CCA detection	Sensitivity 98.5%; specificity 85.7%	Sensitivity 96.3%, specificity 85.4%
Gou, Q. et al (58)	cfDNA for somatic mutations detection	71.4% (20/28)	53.6% (15/28)
Ito, S. et al. (59)	cfDNA for Cancer driver mutations detection	58.8% (10/17)	17.6% (3/17)
Yoshida, M. et al. (63)	miR-21-5p for diagnostic performance	AUC = 0.913	AUC = 0.628
Han, J. Y. et al (110)	ctDNA for somatic mutations detection	80.0% (16/20)	42.9% (6/14)
Driescher, C. et al (111)	cfDNA for Cancer driver mutations detection	96.2%	31.6%

AUC, Area Under the Curve

In summary, bile offers significant advantages for the early screening, precise treatment monitoring, and prognostic evaluation of BTCs due to its direct interaction with tumors. Tumor cells that are shed directly into bile from the mucosal layer can be identified sooner than CTCs that migrate into the bloodstream. Furthermore, the greater concentration of circulating tumor DNA (ctDNA) in bile might improve the effectiveness of detecting early-stage tumors. However, the more challenging and invasive nature of bile collection poses a limitation for its wider application.

## Liquid biopsy for biliary tract cancers: screening and early diagnosis

BTCs continues to pose significant clinical challenges in terms of early and accurate diagnosis. Currently, accurate preoperative evaluation of BTCs largely depends on imaging methods, which have restricted diagnostic efficacy—especially for small, invasive tumors—while tissue biopsy is still a subject of debate, particularly concerning patients eligible for surgical removal (64, 65). The existing standard serum biomarker, CA19-9, does not offer adequate sensitivity and specificity for identifying early-stage diseases, underscoring a critical demand for more dependable non-invasive diagnostic methods (66). This diagnostic challenge has accelerated the discovery of liquid biopsy biomarkers in BTCs. By detecting tumor-derived biomarkers in blood, bile, and other biofluids, these approaches present significant potential for early cancer detection, particularly in high-risk populations.

Research indicates that the quantitative assessment of cfDNA present in blood possesses diagnostic significance. In patients diagnosed with GBC, the concentration of cfDNA is notably elevated compared to healthy individuals (721.18 ng/ml versus 81.15 ng/ml) (67). Liu, Y. et al. developed an AI-based integrated model named GBCseeker, incorporating cfDNA genetic markers, radiomic features, and clinical information. This model demonstrated high accuracy in distinguishing GBC from gallbladder benign lesions, with an AUC of 93.33% in the discovery cohort and 87.76% in an external validation cohort. Furthermore, it reduced surgeons’ diagnostic error rate by 56.24% (68). In a study by He, J. et al. involving bile samples from 48 patients with CCA and 48 patients with gallstones, it was observed that the expression of Syncytin-1 and SLC7A11 in bile cfDNA gradually increased across groups with gallstones, stage I-II CCA, and stage III-IV CCA. Individually, Syncytin-1 and SLC7A11 showed good diagnostic performance (AUC = 0.805 and 0.755, respectively), while their combination with CA19–9 achieved excellent early diagnostic accuracy (AUC = 0.927) (69). Additionally, research has revealed a significant increase in the proportion of mitochondrial DNA (mtDNA) in the plasma of CCA patients. In certain instances, increased levels of mtDNA were detected despite the absence of a significant rise in cfDNA originating from tumors. A predictive model that integrates mtDNA analysis with copy number assessment increased the AUC from 0.73 (when using copy number variation by itself) to 0.81. This indicates that incorporating mtDNA signals alongside additional liquid biopsy biomarkers might boost the sensitivity of cancer detection (70).

Wen et al. discovered a circRNA complex that is specific to CCA within exosomes derived from both bile and serum. The ability to diagnose using biliary exosomal circRNA signatures (AUROC = 0.947) and serum exosomal circRNA signatures (AUROC = 0.861) exceeded the performance of the traditional CA19–9 marker (AUROC = 0.759) (71). Yang, Y. et al. identified and validated a panel of ten differentially expressed circRNA biomarkers in patients with BTCs. They also developed a DNA-based computational framework that can directly translate biomarker levels into diagnostic outcomes, achieving an accuracy of 83% in the detection of BTCs (72). The AnnV^+^CD44v6^+^ exosome subpopulation distinguished BTCs from HCC with 91% sensitivity and 69% specificity. When combined with other exosomal markers and serum Alpha-Fetoprotein (AFP), the differentiation reached 100% sensitivity and specificity (73). Lapitz, A. et al. analyzed serum EVs from patients with primary sclerosing cholangitis (PSC), PSC with CCA, PSC patients who developed CCA during follow-up, non-PSC-related CCA, hepatocellular carcinoma (HCC), and healthy individuals to identify protein biomarkers. They found that EV protein markers showed high accuracy in diagnosing CCA (AUC = 0.909 for CCA vs. PSC; AUC = 0.931 for CCA vs. non-malignant controls) and could differentiate between HCC and iCCA patients (AUC = 0.850) (74). Furthermore, AnnexinV^+^EpCAM^+^ASGPR1^+^ tumor-associated microparticles (taMPs) were significantly elevated in the serum of CCA patients and demonstrated diagnostic capability with both sensitivity and specificity exceeding 78% (75).

Multiple miRNA biomarkers have demonstrated considerable potential. In GBC, miR-1 exhibits the highest sensitivity (85.7%), while miR-21 shows the highest specificity (92.7%); their combination further improves diagnostic accuracy (76). In bile, miR-451a and miR-3619-3p are stably upregulated in patients with BTCs. When used in combination, they demonstrate favorable diagnostic performance (AUC = 0.819), with high expression of miR-3619-3p indicating poorer prognosis (HR = 2.89) (77). Serum miR-1307-3p shows exceptional discriminative ability, with an AUC of 0.98, sensitivity of 98%, and specificity of 85% (78).

Yang, S. et al. developed a bile metabolite-based platform named BileMet, which enables rapid and accurate detection of malignant biliary tract lesions (AUC = 0.891) (79). Zhang, Z. et al. demonstrated that parallel single-cell genomic sequencing of exfoliated tumor cells (Past-Seq) from bile provides robust evidence for malignant diagnosis. This approach achieved 96% sensitivity, 100% specificity, and 100% positive predictive value in diagnosing CCA from bile, significantly outperforming conventional pathological evaluation (56% sensitivity) and copy number alteration (CNA) analysis of bile cfDNA (13% sensitivity) (80). Additionally, Tsai, Y. C. et al. developed a novel liquid biopsy method—the Cancer Cell Affinity Probing and Tracked by Immunoreaction (CAPTURE) assay—for detecting CCA. This technique uses magnetic microbeads to isolate exfoliated tumor cells (ETCs) and CTCs from the blood and bile of CCA patients, and tracks target cell-bead complexes via immunostaining. The results showed outstanding performance in both bile (98.5% sensitivity, 85.7% specificity) and blood (96.3% sensitivity, 85.4% specificity) for CCA detection, and the method can also be applied for dynamic monitoring of treatment response (81).

Individuals diagnosed with PSC face a lifetime risk of developing CCA that can reach 20%, highlighting the critical necessity for efficient monitoring methods within this group (82–86). Additionally, pancreaticobiliary maljunction (PBM) presents a major risk factor for both CCA and GBC, elevating the likelihood of GBC by 38 times in comparison to the general population (87). Moreover, enduring gallstones, persistent cholecystitis, stones in the bile duct, and parasitic infections of the biliary system are recognized as significant risk factors for BTCs (88). Due to the insufficient sensitivity of existing imaging methods and CA19–9 for identifying early-stage BTCs, it is essential to create liquid biopsy-based strategies for ongoing monitoring of individuals at high risk. Liquid biopsy methods are rapidly advancing the early diagnosis of BTCs through diverse biomarkers and innovative technologies. Although individual biomarkers show promise, optimal diagnostic performance is achieved through multi-marker panels, which provide complementary information and enhance early detection of BTCs. With advancements in AI, radiomics has also demonstrated potential in improving early detection of BTCs (89). As the field evolves, the integration of multi-modal liquid biopsy analyses—including ctDNA, CTCs, and EVs—with radiomics and other data streams is expected to further improve diagnostic specificity and sensitivity. This holistic approach may enable truly earlier intervention and improve patient outcomes in BTCs. Although the concept of ctDNA-, CTC-, and EV-based or multimodal liquid biopsy screening is compelling, its application for population-level or high-risk screening in BTC remains investigational, and existing evidence is not yet sufficient to support its routine clinical use.

## Liquid biopsy for biliary tract cancers: precision therapy

BTCs pose significant therapeutic challenges. Although surgical resection followed by adjuvant chemotherapy, such as fluoropyrimidine-based regimens, remains the standard curative-intent approach, the 3-year recurrence rate can reach as high as 80%, underscoring the urgent need for more effective strategies (90–92). In advanced disease, the results of the TOPAZ-1 and KEYNOTE-966 trials indicate that gemcitabine-cisplatin chemotherapy combined with immunotherapy, either with durvalumab or pembrolizumab, has established itself as the new standard of care. However, the observed survival benefit remains modest, with median overall survival rates reported at approximately 12.9 months compared to 11.3 months, and 10.9 months versus 12.7 months, respectively (93–95). This scenario highlights the constraints of a uniform approach, especially because there are no dependable biomarkers available for selecting patients, which are crucial for identifying individuals who are most likely to gain from this therapy. This unmet need has propelled liquid biopsy to the forefront of precision oncology in BTCs (96). Liquid biopsy involves the analysis of cfDNA, CTCs, and EVs in bodily fluids such as blood or bile, enabling the capture of spatial and temporal tumor heterogeneity. It offers a powerful, non-invasive tool for personalized treatment, particularly in cases where traditional tissue biopsy is limited or unfeasible (97).

Predicting and monitoring responses to immunotherapy is of significant clinical importance in the treatment of BTCs. Studies have identified an immunogenic subtype of iCCA in Asian populations, characterized by T-cell exhaustion and neutrophil extracellular traps (NETs). This subtype can be detected by elevated levels of specific exosomal circular RNAs (e.g., circ-PTPN22 and circ-ADAMTS6) in plasma and may exhibit greater sensitivity to immune checkpoint inhibitors (98).

Large-scale studies have shown a strong agreement between ctDNA-based genotyping and tissue genomic profiling in patients with advanced BTCs (99–102). A study by Hwang S et al. indicated that ctDNA effectively reflects tissue genomic features, with strong agreement (sensitivity 84.8%, positive predictive value 79.4%) (99). A comprehensive analysis of 1,671 patients revealed comparable detection rates between cfDNA and tissue for *IDH1* (87%) and *BRAF* V600E (100%) mutations, though discrepancies were observed in *FGFR2* fusions (100). Astier C et al. found complementary value between ctDNA and tissue biopsies—for instance, *FGFR2* mutations were more frequently detected in liquid biopsies, *KRAS* mutations were primarily identified in tissue, and *IDH1* mutations were consistently detected in both (102). Liquid biopsy reliably detects key actionable mutations in BTCs, such as *FGFR2* fusions (present in approximately 10–15% of iCCA cases), *IDH1* mutations (R132), *KRAS* mutations (G12C), *ARID1A* mutations, *ERBB2* amplifications, and less common kinase fusions (*ALK*, *ROS1*, *NTRK*, *RET*), demonstrating high feasibility (103–108). For instance, *FGFR2* fusions can be detected in plasma with a sensitivity of up to 88.9%, and dynamic monitoring of cfDNA can predict response to FGFR inhibitors (FGFRi) and the emergence of resistance earlier than imaging (107). The clinical utility of targeting these alterations has been validated in practice—for example, the detection of an *EML4*-*ALK* rearrangement via liquid biopsy led to a partial response with Ensartinib, while the identification of *ERBB2* amplification enabled effective anti-HER2 therapy (108, 109). Furthermore, bile serves as a rich source of ctDNA in BTCs. Studies have shown that bile ctDNA detects up to 96.2% of pathogenic tumor mutations identified in tissue, significantly outperforming plasma cfDNA. This makes bile-based liquid biopsy a highly reliable alternative when tissue sampling is not feasible (110, 111).

Currently, small-molecule inhibitors targeting mutant IDH1, such as ivosidenib, are being investigated in preclinical and clinical studies as promising therapeutic options for IDH1-mutated CCA (112, 113). Notably, ivosidenib has already received FDA approval for previously treated IDH1-mutant cholangiocarcinoma, demonstrating clinically meaningful improvements in both progression-free and overall survival. *FGFR2* fusions have emerged as one of the most promising precision oncology targets in BTC, with FGFR inhibitors already approved in Europe and the United States for patients with advanced, previously treated iCCA (114). In parallel, HER2-directed therapies continue to expand treatment options for HER2-positive or HER2-mutant BTC. A variety of approaches—including antibody–drug conjugates (ADCs), tyrosine kinase inhibitors, monoclonal antibodies, and bispecific antibodies—are being actively evaluated across clinical settings. Importantly, trastuzumab deruxtecan (T-Dxd) and zanidatamab have received regulatory approval for HER2-positive biliary tract cancers, offering new therapeutic avenues for this molecular subset. These agents have demonstrated promising antitumor activity and improved survival outcomes compared with standard therapies. Furthermore, trastuzumab-based regimens have progressed into first-line clinical trials, underscoring the growing relevance of HER2-targeted strategies in BTC (115).

Beyond initial molecular profiling, liquid biopsy enables real-time monitoring of treatment response and disease progression. A low baseline cfDNA load is associated with better outcomes in targeted therapy, while high cfDNA concentrations often indicate poor prognosis and impaired liver function (107). Changes in ctDNA variant allele frequency (VAF) or specific mutation levels (e.g., *IDH1* R132C monitored via qPCR) correlate with radiological response (RECIST) and can signal disease progression earlier than conventional methods (32, 116).

There is growing interest in developing biomarkers to predict responses to chemotherapy and combined immunochemotherapy regimens (117, 118). Innovative approaches, such as using genome-wide 5-hydroxymethylcytosine (5hmC) profiling of cfDNA to build predictive models, have shown promise. For instance, a ML-based model developed to predict efficacy of the GOLP regimen (Gemcitabine, Oxaliplatin, Lenvatinib, and anti-PD1 antibody) demonstrated high predictive accuracy (AUC = 0.967) (119). Liquid biopsy is also being progressively integrated into next-generation clinical trials. As an example, a Phase II trial is currently using ctDNA analysis to evaluate the efficacy of adjuvant afatinib in postoperative GBC patients with *ERBB* mutations (120). This approach—using liquid biopsy to pre-therapeutically assess potential treatment response—exemplifies the move toward precision oncology tailored to individual patients.

In summary, liquid biopsy is transforming the management of BTCs by overcoming limitations associated with tissue sampling and tumor heterogeneity (96, 121). It provides critical information for molecularly guided patient selection—whether for targeted therapy, immunotherapy, or chemotherapy—enables dynamic monitoring of treatment response, and facilitates the development of novel predictive biomarkers (97, 117, 118, 122). Ultimately, it paves the way for truly individualized precision therapy and improved survival outcomes for BTCs patients.

## Liquid biopsy for biliary tract cancers: prognostic evaluation

Liquid biopsy technologies, through the analysis of biomarkers have demonstrated significant value in prognostic assessment, recurrence monitoring, and treatment response evaluation in BTCs. Numerous studies suggest that fluctuations in ctDNA are closely linked to patient prognosis. Individuals exhibiting a greater maximum variant allele frequency (maxVAF) of somatic mutations within ctDNA demonstrated notably reduced overall survival (OS) and progression-free survival (PFS) following treatment with chemotherapy using gemcitabine and cisplatin (101). Postoperative changes in ctDNA levels can signal iCCA recurrence earlier than CA19-9 (123). Furthermore, ctDNA yield exhibits a strong positive correlation with tumor volume (r = 0.9326, p < 0.0001) (106), and its detection demonstrates a diagnostic accuracy of 90.8% in CCA, with mean sensitivity and specificity of 96.7% and 72.4%, respectively (124). Certain genetic alterations also have prognostic significance; for instance, mutations in the homologous recombination repair pathway identified in bile cfDNA predict unfavorable outcomes for patients with CCA (125).

*KRAS* mutation status serves as an important prognostic biomarker in BTCs. It is detected in approximately 15.6% of CCA cases, with G12D, G12V, and Q61H being the most common subtypes. Patients carrying *KRAS* mutations exhibit significantly shorter overall survival and recurrence-free survival. Detection of *KRAS* G12/G13 mutations in ctDNA shows moderate concordance with tissue testing, demonstrating a sensitivity of 80% and specificity of 93%. Higher mutant allele frequency (MAF) of *KRAS* in ctDNA, especially when combined with elevated CA19–9 levels, is associated with poorer survival outcomes (126).

CTC detection also holds prognostic value. CTC counts correlate with clinical disease progression (127), and in patients with resectable CCA, the presence of CTCs is associated with higher postoperative TNM stage and worse overall survival (128). One study improved CTC detection rates from 19% to 83% by simultaneously analyzing epithelial-type CTCs (eCTCs) and non-classical CTCs (ncCTCs). Patients with a baseline level of ≥1 eCTC per 10 mL of blood had significantly shorter median disease-specific survival (9 months vs. 19 months) (32).

Bile-derived biomarkers demonstrate excellent performance in prognostic evaluation. Mutational analysis of bile cfDNA in patients with primary sclerosing cholangitis (PSC) detected *KRAS*, *GNAS*, and *TP53* mutations in 36.5% of cases, significantly higher than in healthy controls (10%). Among these, 75% of patients later diagnosed with CCA tested positive for bile-derived mutations (66). In miRNA profiling, the level of miR-21-5p in bile demonstrated superior prognostic capability compared to both serum and CA19–9 levels. Elevated expression of miR-21-5p was correlated with reduced overall survival and identified as an independent prognostic factor (63). The high expression of miR-3619-3p significantly correlated with adverse prognosis (HR = 2.89) (77). Furthermore, protein biomarkers contained in serum EVs can be used for prognostic assessment of CCA, providing a non-invasive tool derived from tumor cells for personalized medicine (74).

In summary, liquid biopsy significantly enhances the ability to evaluate prognosis, stratify recurrence risk, and monitor treatment response in BTCs through multi-component, longitudinal dynamic monitoring, demonstrating considerable potential for clinical translation.

## Liquid biopsies for future directions in biliary tract cancers

Recent advancements in liquid biopsy research have introduced innovative approaches to diagnosing and treating cancer, thus propelling the evolution of personalized therapies for tumors. A key focus within the realm of liquid biopsy is the precision in identifying CTCs, cfDNA, and EVs (129). However, each method possesses certain limitations alongside distinct advantages. Indeed, numerous technological advancements have facilitated the clinical translation of promising liquid biopsy biomarkers. In the context of BTCs, blood and bile serve as the two primary biofluid sources. Each offers unique strengths and weaknesses, and their application must be tailored to specific clinical scenarios and technical requirements. Common limitations of ongoing clinical studies include their single-center nature, small sample sizes, and the lack of standardized protocols. The use of liquid biopsy for early diagnosis and treatment guidance in BTCs is still in its infancy, with most studies focusing solely on diagnostic or prognostic objectives. Currently, liquid biopsy technology—particularly in the early screening of BTCs, systematic monitoring of high-risk populations, and the formulation of individualized precision treatment strategies—remains a core yet challenging area urgently requiring breakthroughs.

Research on liquid biopsy in BTCs lags behind that of other cancers, and several promising novel biomarkers have yet to be thoroughly explored in this context. For example, cfDNA methylation and hydroxymethylation analyses have shown encouraging results in early cancer detection, recurrence monitoring, and prediction of treatment response in lung, liver, pancreatic, and colorectal cancers (130–133). The approval for using plasma-derived methylated Septin9 as a colorectal cancer screening tool has been granted by the U.S. Food and Drug Administration (FDA) (134). Furthermore, cfDNA methylation can be utilized for determining tumor origin (46, 135), identifying molecular subtypes (136, 137), and distinguishing primary from metastatic lesions (138). Furthermore, a comprehensive analysis of whole-genome methylation in EV-DNA has addressed two significant technical obstacles—limited DNA quantity and extended fragment length—thereby facilitating its possible use in discovering new cancer biomarker genes (139). A variety of research efforts have shown that cancer modifies platelets via a mechanism referred to as education, which facilitates cancer progression (140–142). Tumor-educated platelets (TEPs) are being recognized more and more for their promising role as a liquid biopsy tool for early cancer detection and prognosis (143, 144). Studies have found that the expression of immune checkpoint proteins in CTCs is a critical factor in predicting response to cancer immunotherapy, which may improve patient selection for immune checkpoint inhibitors (ICIs) (145). Moreover, liquid biopsy research should not focus solely on tumor burden and characteristics; it must also account for local and systemic host responses, as natural tumor progression and therapy resistance often depend on the interplay between the tumor and the host (146).

In the short term, tissue biopsy is unlikely to be fully replaced by liquid biopsy. Nevertheless, technological advancements are continuously refining treatment stratification strategies based on blood, bile, and other biofluids, thereby accelerating the progress of precision medicine in BTCs. There is no—and likely never will be—a single liquid biopsy assay suitable for all clinical scenarios. Test selection should be guided by the specific tumor type, stage, and treatment strategy. Although current research often focuses on individual biomarkers, growing evidence supports that multi-marker panels significantly enhance detection accuracy. Integrating liquid biopsy with radiomics and constructing multimodal analysis models using ML and AI is expected to substantially improve diagnostic performance. The potential of biomarkers in improving early detection, prognostic assessment, and tailored treatment strategies for BTCs is significantly highlighted by these multidimensional methods that integrate advanced technologies. Nevertheless, to effectively translate these technological innovations into clinical applications, additional validation and standardization will be necessary to guarantee their reliability and broad applicability.
